# Factors associated with usability of the EMPOWER-SUSTAIN Global Cardiovascular Risks Self-Management Booklet© among individuals with metabolic syndrome in primary care: a cross-sectional study

**DOI:** 10.1186/s12875-024-02281-z

**Published:** 2024-02-03

**Authors:** Mohamad Abu Zar Abdul-Halim, Noorhida Baharudin, Hasidah Abdul-Hamid, Mohamed-Syarif Mohamed-Yassin, Maryam Hannah Daud, Siti Fatimah Badlishah-Sham, Suraya Abdul-Razak, Anis Safura Ramli

**Affiliations:** 1 Department of Primary Care Medicine, Faculty of Medicine, Universiti Teknologi MARA, Sungai Buloh, Selangor 47000 Malaysia; 2 Institute of Pathology, Laboratory and Forensic Medicine (I-PPerForM), Universiti Teknologi MARA, Sungai Buloh, Selangor 47000 Malaysia; 3 Netherlands Maritime University College (NMUC), Johor Bahru, Johor 80000 Malaysia; 4 Cardio Vascular and Lungs Research Institute (CaVaLRI), Hospital Al-Sultan Abdullah, Universiti Teknologi MARA, Bandar Puncak Alam, Selangor 42300 Malaysia

**Keywords:** Chronic care model, Self-management booklet, Usability, Metabolic syndrome, cardiovascular risk factors

## Abstract

**Background:**

Self-management support has been recognized as one of the most essential elements of the Chronic Care Model (CCM). Inspired by the CCM, the EMPOWER-SUSTAIN Global Cardiovascular Risks Self-Management Booklet^©^ was developed to aid and sustain self-management among patients with metabolic syndrome (MetS) in primary care to prevent cardiovascular complications. However, the usability of this booklet among these patients is not known. Therefore, this study aimed to evaluate the usability of this self-management booklet and identify the factors associated with its usability among patients with MetS in primary care.

**Methods:**

This cross-sectional study was conducted among patients with MetS attending a university primary care clinic in Selangor, Malaysia. The usability score was measured using a previously translated and validated EMPOWER-SUSTAIN Usability Questionnaire (E-SUQ) with a score of > 68 indicating good usability. Multiple logistic regressions determined the factors associated with its usability.

**Results:**

A total of 391 patients participated in this study. More than half (61.4%) had a good usability score of > 68, with a mean (± SD) usability score of 72.8 (± 16.1). Participants with high education levels [secondary education (AOR 2.46, 95% CI 1.04, 5.83) and tertiary education (AOR 2.49, 95% CI 1.04, 5.96)], those who used the booklet at home weekly (AOR 2.94, 95% CI 1.63, 5.33) or daily (AOR 2.73, 95% CI 1.09, 6.85), and those who had social support to use the booklet (AOR 1.64, 95% CI 1.02, 2.64) were significantly associated with good usability of the booklet.

**Conclusions:**

The usability of the EMPOWER-SUSTAIN Global Cardiovascular Risks Self-Management Booklet^©^ was good among patients with MetS in this primary care clinic, which supports its widespread use as a patient empowerment tool. The findings of this study also suggest that it is vital to encourage daily or weekly use of this booklet at home, with the support of family members. The focus should also be given to those with lower education to improve the usability of this booklet for this group of patients.

**Supplementary Information:**

The online version contains supplementary material available at 10.1186/s12875-024-02281-z.

## Background

Cardiovascular diseases (CVDs), namely, ischaemic heart disease and stroke, are the world's leading causes of death, accounting for a combined 15 million fatalities in 2019 [1]. The clustering of CVD risk factors in an individual gives rise to metabolic syndrome (MetS), which is characterized by the presence of central obesity, elevated blood pressure (BP), elevated plasma glucose, and dyslipidaemia [2]. Individuals with MetS are twice as likely as the general population to experience cardiovascular events [2]. MetS is estimated to affect 20–25% of the global adult population [3, 4]. In Malaysia, the prevalence of MetS among adults aged ≥ 30 years was found to be 43.4% [5]. Its rising prevalence is thought to be the driving force behind the CVD epidemic in Malaysia [6], where ischaemic heart disease and stroke have remained the principal causes of death over the last three decades [7].

The management of MetS is complex and requires self-management support, as advocated by the Chronic Care Model (CCM) [8]. This model identifies six interrelated components of a healthcare system, including community resources, health system organization, self-management support, delivery system design, decision support, and clinical information systems, with the primary objective of enhancing outcomes for chronic conditions, such as MetS [8, 9]. Among these components, self-management support has been recognized as one of the most essential elements of the CCM, as it has been proven to improve outcomes for various chronic conditions [10–14].

With this in mind, the EMPOWER-PAR study conducted by Ramli et al. in 2016 developed a self-management support tool named the EMPOWER-PAR Global Cardiovascular Risks Self-Management Booklet© as part of a multifaceted intervention based on the CCM [14]. The EMPOWER-PAR intervention has been proven to be effective in improving glycaemic control among patients with type 2 diabetes mellitus (T2DM) in primary care [14] and has also been proven to be effective in improving adherence to T2DM clinical practice guidelines (CPG) among primary care providers [15]. The booklet has recently been updated to become the EMPOWER-SUSTAIN Global Cardiovascular Risks Self-Management Booklet© with additional content on weight management (diet and exercise) and smoking cessation [15]. The treatment targets were also updated in line with the Malaysian CPG on Primary and Secondary Prevention of Cardiovascular Disease 2017 [16]. Overall, the booklet was designed as an empowerment tool for patients to understand their conditions, risk factors, potential complications, control targets and how to self-manage their cardiovascular risks with the aim of preventing CVD complications.

Usability testing among the end users is a well-known concept in digital health evaluation [17]. Usability is defined as "the extent to which a product can be used by a specific user for a specific goal in a specific context or environment and provides a satisfying experience" [17]. Nevertheless, in the era of exponential growth in digital health, paper-based self-management tools such as this booklet remain relevant in managing patients with chronic conditions as these booklets are more accessible to a broader range of patients, including those who may not be technologically savvy or do not have access to advanced devices [18]. The EMPOWER-SUSTAIN Global Cardiovascular Risks Self-Management Booklet© is currently being distributed among patients with MetS in our primary care clinic so that they can be empowered to actively participate in managing their own health. However, the level of its usability and the factors associated with its usability were not known. Therefore, the objectives of this study were to evaluate the usability of this booklet and identify the factors associated with its usability among patients with MetS in our primary care clinic. The findings would be used to improve the content and acceptability of this booklet among our patients. Identifying the factors associated with its usability would allow us to target specific determinants, e.g., patient groups or characteristics, to improve its usability and plan for future intervention studies.

## Methods

### Study design and population

This cross-sectional study was conducted from September 2020 until June 2023, which included conceptualizing the study, conducting literature reviews, collecting data, analyzing the data and finalizing the manuscript. The study population was patients with MetS attending a university primary care clinic in Selangor, Malaysia.

### Inclusion and exclusion criteria

This study included patients ≥ 18 years old who met all of the following criteria: fulfilled at least 3 out of 5 diagnostic criteria for MetS based on the Joint Interim Statement (JIS) 2009 definition; waist circumference (WC) using South Asian cut-points: male ≥ 90 cm or female ≥ 80 cm, blood pressure (BP): systolic BP ≥ 130 mmHg and/or diastolic BP ≥ 85 mmHg or on treatment for hypertension, fasting plasma glucose (FPG) ≥ 5.6 mmol/L or on treatment for elevated glucose, triglycerides (TG) ≥ 1.7 mmol/L or on treatment for TG, and high-density lipoprotein (HDL-c): male < 1.0 mmol/L or female < 1.3 mmol/L or on treatment for HDL-c [3], attended the university primary care clinic for ≥ 1 year, were able to read and understand the Malay language, had blood investigations FPG and fasting serum lipid (FSL) performed in the last 6 months and used the EMPOWER-SUSTAIN Global Cardiovascular Risks Self-Management Booklet^©^ for at least 6 months.

Patients who fulfilled any of the following criteria were excluded from the study: were receiving renal dialysis, presented with severe hypertension (systolic BP > 180 mmHg and/or diastolic BP > 110 mmHg) at recruitment, had secondary hypertension, were diagnosed with circulatory disorders requiring secondary care over the last year (e.g., acute coronary syndrome, stroke, transient ischaemic attacks, peripheral vascular disease), were pregnant, were diagnosed with malignancy (i.e., all types of cancers), had any form of mental disorders or cognitive impairments that affected the ability to answer the questionnaire (e.g., dementia, learning disability) and were not able to give written informed consent.

### Variable definition

Household income per month (in Malaysian Ringgit [MYR]) was defined based on the Report of Household Income and Basic Amenities Survey 2019 by the Department of Statistics, Malaysia, as per the following: Bottom 40% (B40) < MYR4,850, Middle 40% (M40) MYR4,850–10,959, and Top 20% (T20) > MYR10,959 [19]. Active smokers were defined as those who currently smoke or have smoked any tobacco products within one year. Previous smokers were those who had stopped smoking for more than a year, and nonsmokers were defined as those who had never smoked. Body mass index (BMI) was defined based on the proposed classification of weight by BMI in adult Asians by the World Health Organization (WHO) as per the following: underweight < 18.5 kg/m^2^, normal 18.5–22.9 kg/m^2^, overweight 23–24.9 kg/m^2^, obese I 25–29.9 kg/m^2^, obese II ≥ 30 kg/m^2^ [20]. Hypertension (HTN) and diabetes mellitus (DM) were defined based on their clinician’s diagnosis and/or if patients were taking any antihypertensive or antidiabetic treatment (oral hypoglycemic agent, insulin), respectively.

In this study, the treatment targets for the participants were defined according to the Malaysian CPG on the Management of Type 2 Diabetes Mellitus (6th Edition) [21] and the CPG on the Management of Primary and Secondary Prevention of Cardiovascular Diseases [16], whereby BP of < 140/80 mmHg for DM patients and < 140/90 mmHg for non-DM patients, HDL-c of > 1.0 mmol/L for males and > 1.2 mmol/L for females, TG of ≤ 1.7 mmol/L, and low-density lipoprotein cholesterol (LDL-c) of ≤ 2.6 mmol/L were defined as well controlled [16, 21].

### Study tool

The tool used to measure usability in this study was the EMPOWER-SUSTAIN Usability Questionnaire (E-SUQ), which is valid, reliable, and stable for measuring the usability of a self-management booklet among patients with MetS in primary care [22]. The E-SUQ is a 10-item questionnaire in the Malay language adapted from the System Usability Scale (SUS) questionnaire; therefore, its scoring system is similar to that of the SUS [23, 24]. The E-SUQ is provided in the Additional file 1. The score was calculated using a 5-point Likert scale ranging from 1 (strongly disagree) to 5 (strongly agree). The item score on the positively phrased statements, i.e., odd-numbered questions, was subtracted by 1 (x−1), and the item score on the negatively phrased statements, i.e., even-numbered questions, was calculated by subtracting the score from 5 (5−x) [25]. The overall score was computed as the sum of all item scores multiplied by 2.5, which gave an overall score that ranged from 0 (extremely poor usability) to 100 (excellent usability). A score of > 68 indicates good usability, as recommended by the original author [23]. Permission to use the E-SUQ was obtained from the questionnaire developer.

### Sampling frame and distribution of the booklet

**Fig. 1:**
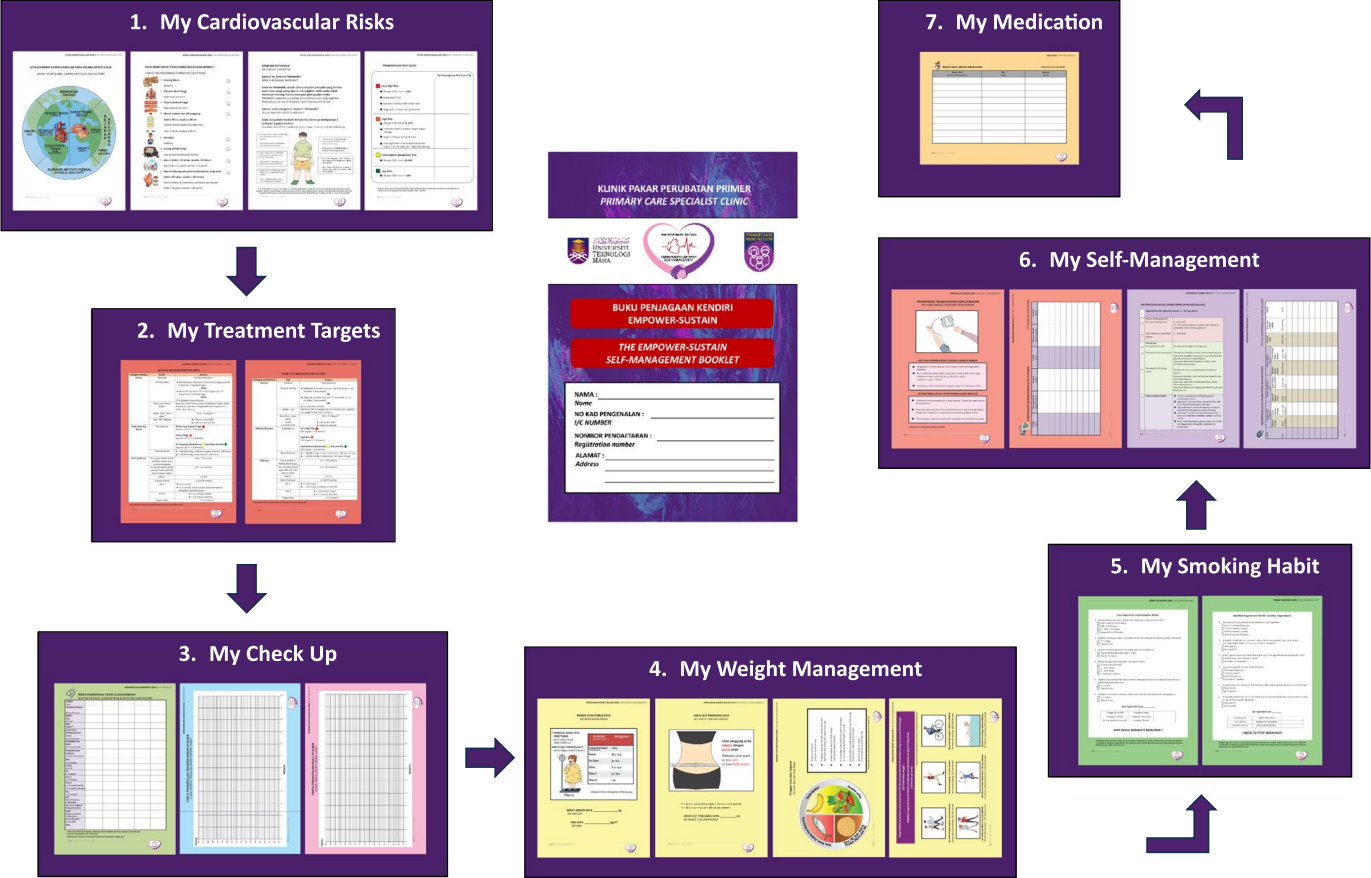
The EMPOWER-SUSTAIN Global Cardiovascular Risks Self-Management Booklet©

The sampling frame was approximately 2000 patients with MetS attending the university primary care clinic. The EMPOWER-SUSTAIN Global Cardiovascular Risks Self-Management Booklet© was distributed to all patients with MetS attending this clinic between May 2020 and September 2021. The patients were counselled by the researcher to use the booklet to monitor their health at home and to bring the booklet to each follow-up appointment with their doctors. This is to ensure that patients had the booklet for at least six months before the study's recruitment and the collection of usability data. This booklet contained seven sections, as shown in Fig. 1.

### Study conduct: recruitment and data collection procedures

Patient recruitment and data collection were conducted from July 2021 to September 2022. A trained research assistant recruited patients and collected the data to maintain a consistent method. Patients who had the booklet were consecutively identified on the day of their follow-up clinic appointment. They were approached in the nurse assessment room, given the study information sheet (containing background, purpose, benefit, study procedure, confidentiality status and contact information) and were invited to participate. Patients who verbally agreed to participate were screened for eligibility following the inclusion and exclusion criteria. Trained nurses measured WC, BMI and BP. The patient's medical history and blood investigations, which were required to diagnose MetS (FPG and FSL), were retrieved from the electronic medical record (EMR). Patients who fulfilled the eligibility criteria were recruited into the study, and written informed consent was obtained. Sociodemographic data, which included age, gender, marital status, education level, employment status, income and clinical factors such as self-reported health status and smoking status, were collected by trained research assistants.

### Questionnaire administration

The E-SUQ was self-administered by the participants. Prior to the administration of the questionnaire, clear verbal instructions on how to complete the questionnaire were given to the participants. Participants were reminded to complete the questionnaire in approximately 20 minutes without referring to notes or family members. They were free to ask for clarification from the researcher at any time should any query arise. Once the questionnaire was completed, participants were requested to return it to the researcher, who checked it for completeness.

### Sample size calculation

The sample size was calculated using OpenEpi, Version 3.01 [26], an open-source online calculator software using the Single Proportion Formula. As we did not find any published literature measuring the usability of a self-management booklet, the sample size for this study was calculated based on a study measuring the usability of a mobile app using the SUS questionnaire, i-Predi, where 65% of participants rated the app as having good usability [27]. With reference to this proportion of good usability, taking an α value of 0.05 with an absolute precision of 5%, the minimum required number of participants was 350. Considering a nonresponder and ineligibility rate of 10%, the study aimed to approach 385 patients.

### Statistical analysis

The latest IBM SPSS Statistics Program Version 28 was used for data entry and statistical analysis [28]. The data quality was evaluated based on the percentage of missing data, and the formula recommended by the SUS author [25] was used to calculate the mean E-SUQ score. For categorical data, descriptive analysis was presented as frequency and percentage. Normally distributed continuous data were expressed as the mean with standard deviation (± SD), and nonnormally distributed data were expressed as the median with interquartile range (IQR). Simple logistic regression (SLogR) was performed to determine the association between the independent variables and the dependent variable, i.e., the  proportion of participants with good usability of the booklet (E-SUQ score value of > 68). Variables with a *P* value of < 0.25 from the SLogR were included in the multiple logistic regressions (MLogR) to determine the factors associated with good usability. A *P* value of < 0.05 was considered significant for MLogR.

## Results

**Fig. 2:**
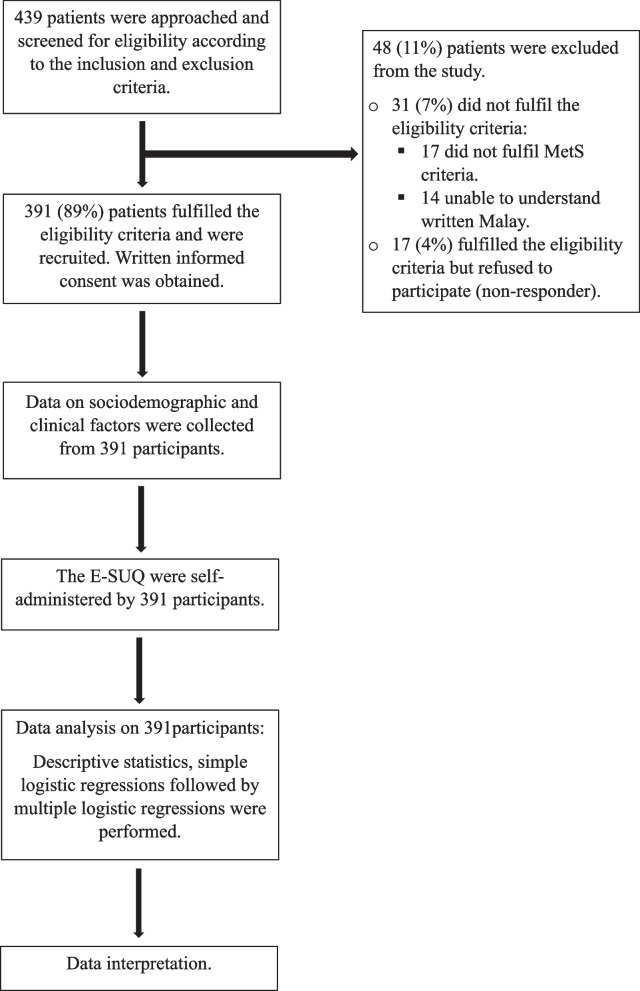
Flowchart of the study

A total of 439 patients were approached and invited to participate. Of these, 391 (89%) patients were eligible and recruited into the study. The flowchart for the conduct of the study is shown in Fig. 2.

### Sociodemographic characteristics of the participants

Table 1 summarizes the sociodemographic characteristics of the participants in this study. The mean (± SD) age was 61.7 (± 8.9), ranging between 27 and 80 years old. Of the 391 participants, the majority were Malays (86.4%), married (87.0%), had a tertiary education (52.2%), were pensioners (50.6%), had < 4 household members (46.5%), and came from the bottom 40% household income group (60.6%). The gender distribution was almost equal (males 50.9% vs. females 49.1%).

**Table 1:** Sociodemographic characteristics of the participants, *N* = 391

*Sociodemographic characteristic*	
***Age (years)**** [mean (SD)]*	61.7 (8.9)
***Gender**** (n, %)*	
Male	199 (50.9)
Female	192 (49.1)
***Ethnicity**** (n, %)*	
Malay	338 (86.4)
Chinese	25 (6.4)
Indian	24 (6.1)
Others	4 (1)
***Marital status**** (n, %)*	
Single	12 (3.1)
Widow/Widower	28 (7.2)
Divorced	11 (2.8)
Married	340 (87.0)
***Educational level**** (n, %)*	
No formal education	3 (0.8)
Primary school	30 (7.7)
Secondary school	154 (39.4)
Tertiary education	204 (52.2)
***Occupation**** (n, %)*	
Housewives/Unemployed	77 (19.7)
Pensioner	198 (50.6)
Skilled/Semiskilled	68 (17.4)
Managerial/Professional	48 (12.3)
***Number of household members**** (n, %)*	
< 4	182 (46.5)
4–6	170 (43.5)
> 6	39 (10.0)
***Household income groups**** (n, %)*	
B40 (< MYR4,850)	237 (60.6)
M40 (MYR4,850–10,959)	124 (31.7)
T20 (> MYR10,959)	30 (7.7)

### Clinical characteristics of participants

Table 2 summarizes the clinical characteristics of the participants. Out of 391 participants, the majority never smoked cigarettes (75.7%), self-rated to have good health status (57.0%), were obese class II (45.5%), had abdominal obesity (85.2%), had DM (66.2%), had HTN (89.5%), and had uncontrolled systolic blood pressure (51.2%). The majority of them had controlled diastolic blood pressure (69.6%), controlled LDL-c (67.5%), controlled HDL-c (71.4%), controlled TG (72.6%), had all 5 MetS components (62.7%), and were on 4–6 medications (48.6%). The mean FPG was 6.54 (SD ± 1.85), and the mean total cholesterol (TC) was 4.46 (SD ± 1.05).

**Table 2:** Clinical characteristics of the participants, *N* = 391

*Clinical Characteristics*	
***Smoking status**** (n, %)*	
Active smoker	30 (7.7)
Ex-smoker	65 (16.6)
Never smoked	296 (75.7)
***Self-rated health status**** (n, %)*	
Very bad	0 (0.0)
Not good	12 (3.1)
Moderate	125 (32.0)
Good	223 (57.0)
Very good	31 (7.9)
***Body mass index**** (n, %)*	
Underweight (< 18.5 kg/m^2^)	1 (0.3)
Normal (18.5–22.9 kg/m^2^)	39 (10.0)
Overweight (23–24.9 kg/m^2^)	36 (9.2)
Obese I (25–29.9 kg/m^2^)	137 (35.0)
Obese II (≥ 30 kg/m^2^)	178 (45.5)
***Abdominal obesity (waist circumference male* ≥ *90 cm, female* ≥ *80 cm)**** (n, %)*	
Yes	333 (85.2)
No	58 (14.8)
**Diabetes mellitus** (n, %)	
Yes	259 (66.2)
No	132 (33.8)
***Fasting plasma glucose (mmol/L)**** [mean (*± *SD)]*	6.54 (± 1.85)
***Hypertension**** (n, %)*	
Yes	350 (89.5)
No	41 (10.5)
***Achieved systolic blood pressure control (*< *140 mmHg)**** (n, %)*	
Yes	191 (48.8)
No	200 (51.2)
***Achieved diastolic blood pressure control (diabetes mellitus*** ** < *80 mmHg, nondiabetes mellitus* < *90 mmHg)**** (n, %)*	
Yes	272 (69.6)
No	119 (30.4)
***Total cholesterol (mmol/L)**** [mean (*± *SD)]*	4.46 (± 1.05)
***Abnormal high-density lipoprotein cholesterol, (male* ≤ *1.0 mmol/L, female* ≤ *1.2 mmol/L)**** (n, %)*	
Yes	112 (28.6)
No	279 (71.4)
***Elevated triglycerides, (*> *1.7 mmol/L)**** (n, %)*	
Yes	107 (27.4)
No	284 (72.6)
***Elevated low-density lipoprotein cholesterol, (*> *2.6 mmol/L)**** (n, %)*	
Yes	127 (32.5)
No	264 (67.5)
***Number of metabolic syndrome components**** (n, %)*	
5 out of 5	245 (62.7)
4 out of 5	112 (28.6)
3 out of 5	34 (8.7)
***Number of medications**** (n, %)*	
> 6	54 (13.8)
4–6	190 (48.6)
< 4	147 (37.6)

### Mean usability score and distribution of usability levels

**Fig. 3:**
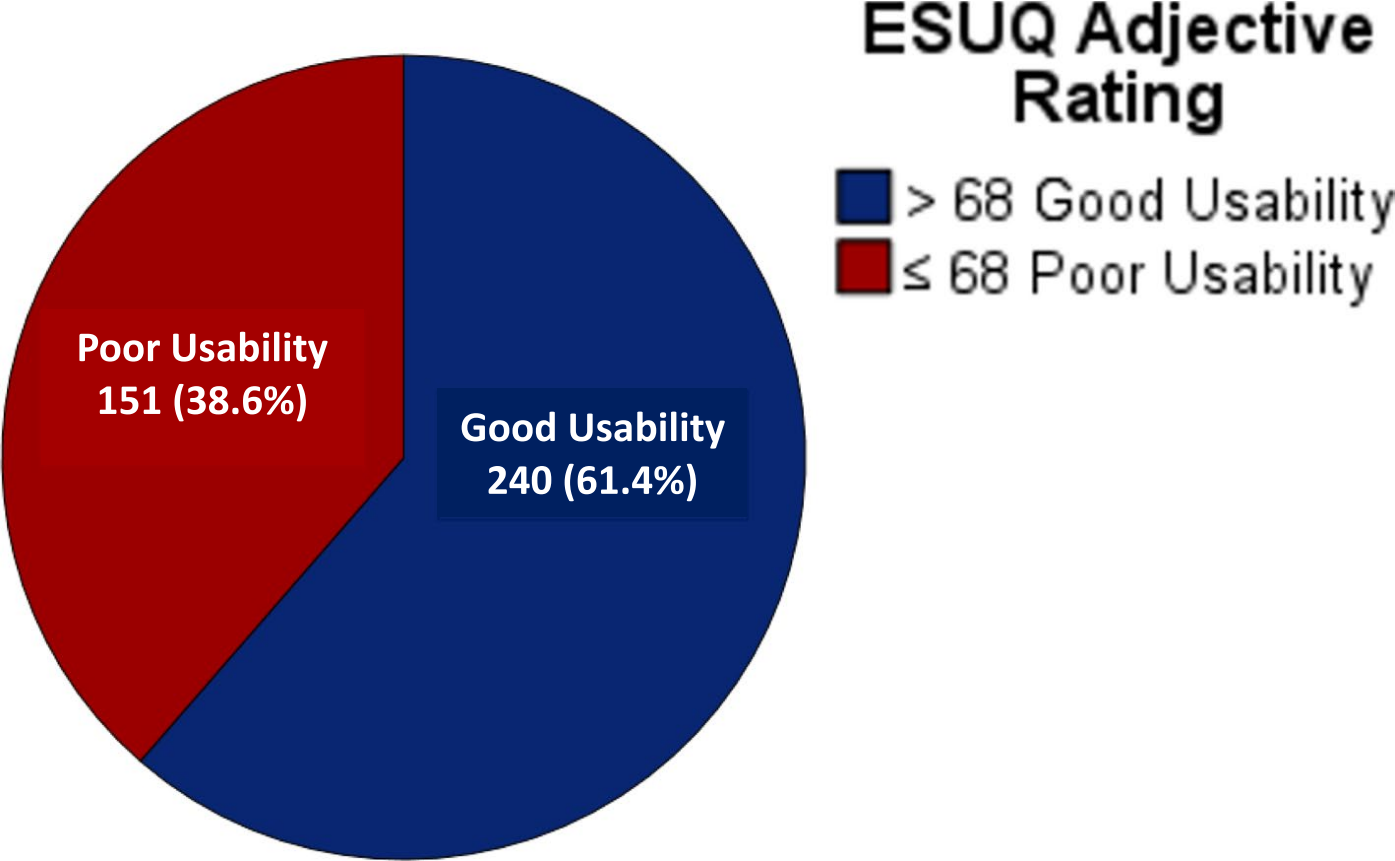
Distribution of participants with good and poor usability levels

Out of 391 participants, more than half (61.4%) of the participants reported a good usability score (score of > 68), as measured by the E-SUQ, as shown in Fig. 3. The mean usability score was 72.8 (SD ± 16.1).

### Distribution of participants’ responses toward the features of the EMPOWER-SUSTAIN Global Cardiovascular Risks Self-Management Booklet©

Table 3 summarizes the distribution of participants’ responses regarding the features of the booklet. The majority of the participants concurred that they liked the physical appearance of the booklet in terms of size, design and typography. They also agreed that all seven sections of the booklet were easy to understand.

**Table 3:** Distribution of participants’ responses to the features of the EMPOWER-SUSTAIN Global Cardiovascular Risks Self-Management Booklet^©^, *N* = 391

*Participants responses*	
***Do you like the size of this booklet? (n, %)***	
Yes	340 (87.0)
No	51 (13.0)
***Do you like the design of this booklet? (n, %)***	
Yes	379 (96.9)
No	12 (3.1)
***Do you like the colour of this booklet? (n, %)***	
Yes	382 (97.7)
No	9 (2.3)
***Is the font size easy to read? (n, %)***	
Yes	376 (96.2)
No	15 (3.8)
***Is the overall content of the booklet well-organized and easy to follow? (n, %)***	
Yes	371 (94.9)
No	20 (5.1)
***Is the content relating to the ‘Cardiovascular Risks’ section easy to understand? (n, %)***	
Yes	347 (88.7)
No	44 (11.3)
***Is the content relating to the ‘Treatment Targets’ section easy to understand? (n, %)***	
Yes	361 (92.3)
No	30 (7.7)
***Is the content relating to the ‘Check-Up’ section easy to understand? (n, %)***	
Yes	369 (94.4)
No	22 (5.6)
***Is the content relating to the ‘Weight Management’ section easy to understand? (n, %)***	
Yes	364 (93.1)
No	27 (6.9)
***Is the content relating to the ‘Smoking Habit’ section easy to understand? (n, %)***	
Yes	277 (70.8)
No	114 (29.2)
***Is the content relating to the ‘Self-Management’ section easy to understand? (n, %)***	
Yes	370 (94.6)
No	5.4 (21)
***Is the content relating to the ‘Medications’ section easy to understand? (n, %)***	
Yes	350 (89.5)
No	41 (10.5)

### Distribution of participants’ responses toward doctors’ support in using the EMPOWER-SUSTAIN Global Cardiovascular Risks Self-Management Booklet©

Table 4 summarizes the distribution of participants’ responses toward doctors’ support in using the EMPOWER-SUSTAIN Global Cardiovascular Risks Self-Management Booklet^©^. More than half of the participants had one treating doctor (61.6%), and 88.5% of the participants were given explanations by the doctors on how to use the booklet, especially on their treatment targets (96.8%), investigation results (95.1%), home blood pressure monitoring (95.5%) and self-monitoring of blood sugar (94.4%).

**Table 4:** Distribution of participants’ responses to doctors’ support in using the EMPOWER-SUSTAIN Global Cardiovascular Risks Self-Management Booklet^©^

***Participants responses, N = 391***	
***Number of treating doctor(s) (n, %)***	
1	241 (61.6)
2–3	122 (31.2)
> 3	28 (7.2)
***Explanation by doctor(s) on how to use the booklet (n, %)***	
Yes	346 (88.5)
No	45 (11.5)
***Participants who received an explanation from the doctors on how to use the booklet, n = 346***	
***Doctor(s) explained regarding cardiovascular risks using the booklet (n, %)***	
Yes	287 (82.9)
No	59 (17.1)
***Doctor(s) explained the treatment targets using the booklet (n, %)***	
Yes	335 (96.8)
No	11 (3.2)
***Doctor(s) explained the investigation results using the booklet (n,%)***	
Yes	329 (95.1)
No	17 (4.9.9)
***Doctor(s) explained regarding healthy eating and/or exercise using the booklet (n, %)***	
Yes	304 (87.9)
No	42 (12.1)
***Doctor(s) explained regarding home blood pressure monitoring using the booklet***^**a**^*** (n, %)***	
Yes	296 (95.5)
No	14 (4.5)
***Doctor(s) explained regarding self-monitoring of blood sugar using the booklet***^**b**^*** (n, %)***	
Yes	221 (94.4)
No	13 (5.6)
***Doctor(s) explained regarding medications using the booklet (n, %)***	
Yes	286 (82.7)
No	60 (17.3)

^a^Hypertension patients only, n = 310, ^b^Diabetes mellitus patients only, n = 234

### Distribution of participants’ responses toward using the EMPOWER-SUSTAIN Global Cardiovascular Risks Self-Management Booklet© at Home

Table 5 summarizes the distribution of participants’ responses toward using the EMPOWER-SUSTAIN Global Cardiovascular Risks Self-Management Booklet^©^ at home. Majority of the participants had utilized the booklet at home (75.7%), and 39.6% used the booklet weekly. Most patients utilized the booklet primarily to self-monitor their blood sugar (93.9%), followed by blood pressure (93.3%).

**Table 5:** Distribution of participants’ responses toward using the EMPOWER-SUSTAIN Global Cardiovascular Risks Self-Management Booklet© at home

***Participants responses, N = 391***	
***Using the booklet at home**** (n, %)*	
Yes	296 (75.7)
No	95 (24.3)
***Frequency of using the booklet at home (n, %)***	
None	95 (24.3)
Monthly	108 (27.6)
Weekly	155 (39.6)
Daily	33 (8.4)
***Participants who used the booklet at home, n = 296***	
***Using the booklet at home to understand their cardiovascular risks (n, %)***	
Yes	226 (76.4)
No	70 (23.6)
***Using the booklet at home to understand their treatment targets (n, %)***	
Yes	271 (91.6)
No	25 (8.4)
***Using the booklet at home to understand their investigations results (n, %)***	
Yes	267 (90.2)
No	29 (9.8)
***Using the booklet at home to understand healthy eating and exercise (n, %)***	
Yes	227 (76.7)
No	69 (23.3)
***Using the booklet at home to self-monitor blood pressure***^**a**^*** (n, %)***	
Yes	252 (93.3)
No	18 (6.7)
***Using the booklet at home to self-monitor blood sugar***^**b**^*** (n, %)***	
Yes	185 (93.9)
No	12 (6.1)
***Using the booklet at home to understand their medications (n, %)***	
Yes	196 (66.2)
No	100 (33.8)

^a^Hypertension patients only, n = 270, bDiabetes mellitus patients only, n = 197

### Distribution of participants’ responses toward social support in using the EMPOWER-SUSTAIN Global Cardiovascular Risks Self-Management Booklet©

**Fig. 4:**
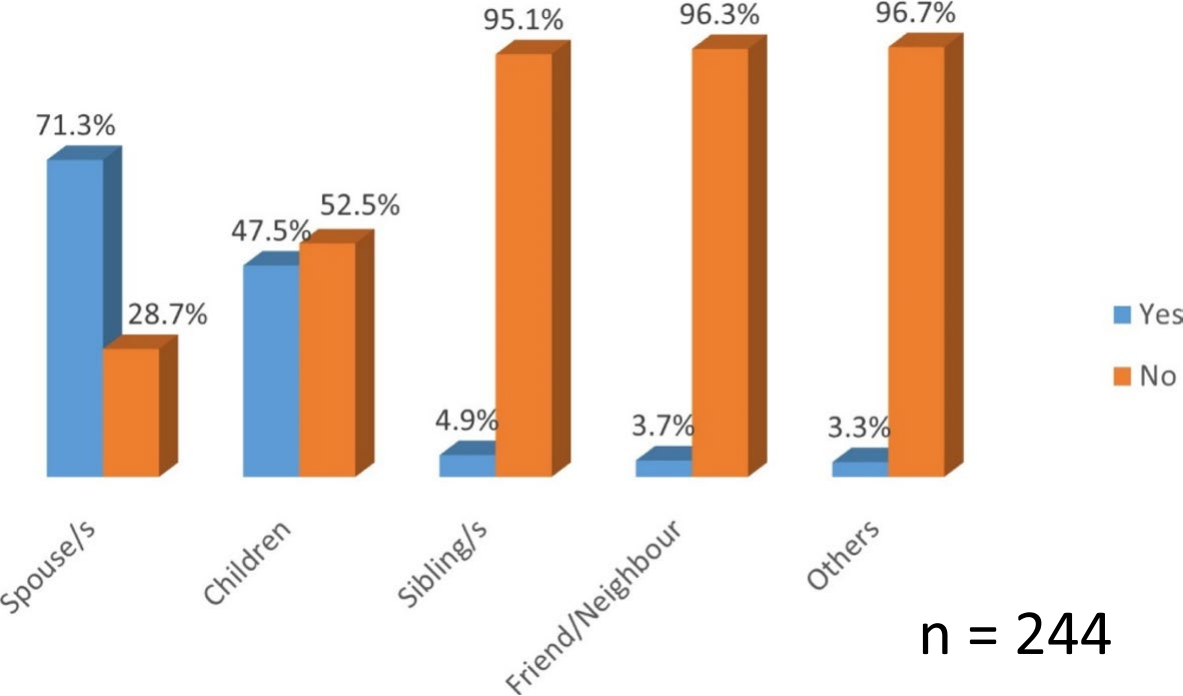
Distributions of social support in using the booklet at home

Out of 391 patients, 244 (62.4%) received social support from their family members and friends in using the EMPOWER-SUSTAIN Global Cardiovascular Risks Self-Management Booklet^©^. Of those who received social support to use the booklet (n = 244), the majority of them received it from their spouses (71.3%) (Fig. 4).

### Factors associated with usability of the EMPOWER-SUSTAIN Global Cardiovascular Risks Self-Management Booklet©

Table 6 shows the results of the logistic regressions. A total of 15 variables had a *P* value of < 0.25 in the SLogR, and these variables were then included in the MLogR. These were being Malay (*P* = 0.050), being married (*P* = 0.026), having secondary education level (*P* = 0.011), having tertiary education level (*P* = 0.002), being in the M40 household income group (*P* = 0.022), being in the T20 household income group (*P* = 0.077), having moderate self-reported health status (*P* = 0.125), having good self-reported health status (*P* = 0.202), being overweight (*P* = 0.067), being obese class I (*P* = 0.117), having explanations given by doctor/s on how to use the booklet (*P* = 0.033), using the booklet at home monthly (*P* = 0.001), using the booklet at home weekly (*P* =  < 0.001), using the booklet at home daily (*P* = 0.004), and having social support to use the booklet (*P* = 0.001).

**Table 6:** Factors associated with usability of the EMPOWER-SUSTAIN Global Cardiovascular Risks Self-Management Booklet^©^

***Variables***	***Simple Logistic Regressions***				***Multiple Logistic Regressions***			
	***B (SE)***	***Wald (df)***	***Crude OR*** ***(95% CI)***	***P value***	***Adjusted B (SE)***	***Wald (df)***	***Adjusted OR*** ***(95% CI)***	***P value***
***Age (years)***	-0.01 (0.01)	1.33 (1)	0.99 (0.96, 1.01)	0.250	-	-	-	-
***Gender***								
Male			1					
Female	-0.04 (0.21)	0.03 (1)	0.96 (0.64, 1.45)	0.860	-	-	-	-
***Race***								
Non-Malay			1				1	
Malay	0.58 (0.30)	3.86 (1)	1.79 (1.00, 3.21)	0.05	0.32 (0.33)	0.93 (1)	1.38 (0.72, 2.63)	0.336
***Marital status***								
Unmarried (Single/Widow/Divorced)			1				1	
Married	0.67 (0.30)	4.95 (1)	1.96 (1.08, 3.54)	**0.026**	0.46 (0.35)	1.76 (1)	1.58 (0.80, 3.11)	0.185
***Education level***								
Low education (No formal education/Primary school)			1				1	
Secondary education	1.01 (0.40)	6.43 (1)	2.74 (1.26, 5.98)	**0.011**	0.90 (0.44)	4.17 (1)	2.46 (1.04, 5.83)	**0.041**
Tertiary education	1.21 (0.39)	9.57 (1)	3.35 (1.56, 7.21)	**0.002**	0.91 (0.45)	4.20 (1)	2.49 (1.04, 5.96)	**0.040**
***Occupation***								
Housewives/Unemployed			1					
Pensioner	-0.03 (0.28)	0.01 (1)	0.97 (0.56, 1.67)	0.912	-	-	-	-
Skilled/Semiskilled	-0.15 (0.34)	0.19 (1)	0.86 (0.44, 1.68)	0.666	-	-	-	-
Managerial/Professional	0.01 (0.38)	0.0 (1)	1.01 (0.48, 2.12)	0.985	-	-	-	-
***Number of household members***								
< 4			1					
4–6	-0.04 (0.22)	0.03 (1)	0.96 (0.63, 1.48)	0.861	-	-	-	-
> 6	-0.13 (0.36)	0.13 (1)	0.88 (0.43, 1.78)	0.717	-	-	-	-
***Household income groups***								
B40: < MYR4,850			1				1	
M40: MYR4,850–10,959	0.53 (0.23)	5.21 (1)	1.70 (1.08, 2.69)	**0.022**	0.41 (0.27)	2.39 (1)	1.51 (0.90, 2.54)	0.122
T20: > MYR10,959	0.77 (0.43)	3.13 (1)	2.15 (0.92, 5.03)	0.077	0.58 (0.48)	1.46 (1)	1.78 (0.70, 4.54)	0.227
***Smoking status***								
Active smoker			1					
Ex-smoker	0.34 (0.45)	0.57 (1)	1.40 (0.58, 3.36)	0.451	-	-	-	-
Never smoked	0.36 (0.39)	0.89 (1)	1.44 (0.68, 3.06)	0.346	-	-	-	-
***Self-rated health status***								
Very bad/Not good			1				1	
Moderate	-1.23 (0.80)	2.35 (1)	0.29 (0.06, 1.41)	0.125	-1.56 (0.91)	2.98 (1)	0.21 (0.04, 1.24)	0.085
Good	-1.01 (0.79)	1.63 (1)	0.36 (0.08, 1.72)	0.202	-1.44 (0.90)	2.56 (1)	0.24 (0.04, 1.39)	0.110
Very good	-0.76 (0.87)	0.77 (1)	0.47 (0.09, 2.57)	0.382	-0.92 (0.98)	0.89 (1)	0.40 (0.06, 2.72)	0.346
***Body mass index, (kg/m***^***2***^***)***								
Obese II (≥ 30)			1				1	
Obese I (25–29.9)	-0.37 (0.24)	2.45 (1)	0.69 (0.44, 1.10)	0.117	-0.39 (0.26)	2.35 (1)	0.68 (0.41, 1.12)	0.126
Overweight (23–24.9)	-0.68 (0.37)	3.36 (1)	0.51 (0.25, 1.05)	0.067	-0.60 (0.41)	2.07 (1)	0.55 (0.25, 1.24)	0.151
Underweight/Normal (< 23)	-0.17 (0.36)	0.21 (1)	0.85 (0.42, 1.73)	0.648	0.08 (0.41)	0.04 (1)	1.09 (0.49, 2.41)	0.836
***Abdominal obesity (waist circumference male* ≥ *90 cm, female* ≥ *80 cm)***								
Yes			1					
No	0.12 (0.30)	0.17 (1)	1.13 (0.63, 2.01)	0.683	-	-	-	-
***Diabetes mellitus***								
Yes			1					
No	0.10 (0.22)	0.19 (1)	1.10 (0.71, 1.70)	0.664	-	-	-	-
***Fasting plasma glucose (mmol/L)***	0.0 (0.07)	0.0 (1)	1.00 (0.90, 1.12)	0.988	-	-	-	-
***Hypertension***								
Yes			1					
No	-0.13 (0.34)	0.16 (1)	0.88 (0.45, 1.69)	0.693	-	-	-	-
***Achieved systolic blood pressure control*** ***(*< *140 mmHg)***								
No			1					
Yes	0.12 (0.21)	0.33 (1)	1.13 (0.75, 1.69)	0.566	-	-	-	-
***Achieved diastolic blood pressure control (diabetes mellitus* < *80 mmHg, nondiabetes mellitus* < *90 mmHg)***								
No			1					
Yes	0.10 (0.23)	0.21 (1)	1.11 (0.71, 1.72)	0.645	-	-	-	-
***Total cholesterol (mmol/L)***	-0.09 (0.10)	0.77 (1)	0.92 (0.76, 1.11)	0.379	-	-	-	-
***Abnormal high-density lipoprotein cholesterol, (male* ≤ *1.0 mmol/L, female* ≤ *1.2 mmol/L)***								
Yes			1					
No	-0.07 (0.23)	0.08 (1)	0.94 (0.60, 1.47)	0.773	-	-	-	-
***Elevated triglycerides, (*> *1.7 mmol/L)***								
Yes			1					
No	-0.02 (0.23)	0.01 (1)	0.98 (0.62, 1.55)	0.940	-	-	-	-
***Elevated low-density lipoprotein cholesterol, (*> *2.6 mmol/L)***								
Yes			1					
No	-0.0 (0.22)	0.0 (1)	1.00 (0.65, 1.54)	0.992	-	-	-	-
***Number of metabolic syndrome components***								
5 out of 5			1					
4 out of 5	0.01 (0.23)	0.0 (1)	1.01 (0.64, 1.60)	0.956	-	-	-	-
3 out of 5	0.42 (0.13)	1.29 (1)	1.57 (0.72, 3.43)	0.256	-	-	-	-
***Total number of medications***								
> 6			1					
4–6	-0.0 (0.32)	0.0 (1)	1.00 (0.54, 1.85)	0.994	-	-	-	-
< 4	0.03 (0.33)	0.01 (1)	1.03 (0.55, 1.96)	0.918	-	-	-	-
***Number of treating doctor/s***								
1			1					
2–3	-0.22 (0.23)	0.96 (1)	0.80 (0.51, 1.25)	0.328	-	-	-	-
> 3	-0.27 (0.41)	0.43 (1)	0.77 (0.35, 1.70)	0.512	-	-	-	-
***Explanation by doctor/s on how to use the booklet***								
No			1				1	
Yes	0.68 (0.32)	4.54 (1)	1.97 (1.06, 3.68)	**0.033**	0.40 (0.36)	1.24 (1)	1.49 (0.74, 2.99)	0.266
***Frequency of using the booklet at home***								
None			1				1	
Monthly	0.94 (0.29)	10.49 (1)	2.55 (1.45, 4.49)	**0.001**	0.59 (0.32)	3.39 (1)	1.80 (0.96, 3.35)	0.065
Weekly	1.33 (0.28)	23.43 (1)	3.78 (2.21, 6.49)	**< 0.001**	1.08 (0.30)	12.72 (1)	2.94 (1.63, 5.33)	**< 0.001**
Daily	1.24 (0.43)	8.19 (1)	3.45 (1.48, 8.06)	**0.004**	1.01 (0.47)	4.60 (1)	2.73 (1.09, 6.85)	**0.032**
***Had social support to use the booklet***								
No			1				1	
Yes	0.70 (0.21)	10.53 (1)	2.01 (1.32, 3.05)	**0.001**	0.49 (0.24)	4.13 (1)	1.64 (1.02, 2.64)	**0.042**

1 = Reference group. Emboldened: Significant at *P* < 0.05

Nagelkerke R^2^ = 0.180. Model fitness was checked using the Hosmer and Lemeshow test (*P* > 0.05). There were no significant interactions or multicollinearity problems (variance inflation factor < 5). All assumptions were met. Sensitivity 86.3%, specificity 45.7%

From the MLogR, five variables were found to be significantly associated with the good usability of the EMPOWER-SUSTAIN Global Cardiovascular Risks Self-Management Booklet^©^ (*P* value < 0.05), namely, having secondary education level, tertiary education level, daily use of the booklet at home, weekly use of the booklet at home, and having social support to use the booklet. For those who had secondary education (AOR 2.46, 95% CI 1.04, 5.83) and tertiary education (AOR 2.49, 95% CI 1.04, 5.96), the odds of finding the booklet usable were more than double compared to those with low education levels (no formal education and primary school). Those who used the booklet weekly (AOR 2.94, 95% CI 1.63, 5.33) and daily (AOR 2.73, 95% CI 1.09, 6.85) at home had almost three times the odds of finding the booklet usable compared with those who did not use the booklet at home. For those who had social support to use the booklet, the odds of finding the booklet usable were almost twice as high (AOR 1.64, 95% CI 1.02, 2.64) compared with those who did not have social support. The other variables, i.e., being Malay, being married, having at least middle-class household income, having moderate and good self-reported health, being overweight and obese I, and having an explanation given by the doctor(s) regarding how to use the booklet, were adjusted as confounders.

## Discussion

To the best of our knowledge, our study was the first in Malaysia to measure the usability of a self-management booklet in primary care. Most of the published research has evaluated the usability of electronic health interventions, such as mobile applications [18, 27, 29–33]. More than half (61.4%) of the patients with MetS in our study had a good usability score of > 68 as measured by the E-SUQ. The mean usability score among our patients was 72.8 (SD ± 16.1), which is comparable to the mean usability score found in our previous field-testing validation study (77.3 [SD ± 13.8]) [22]. This has proven that the usability of the EMPOWER-SUSTAIN Global Cardiovascular Risks Self-Management Booklet^©^ among patients with MetS in our primary care clinic is good [23]. However, a direct comparison with other studies evaluating the usability of a self-management booklet is difficult because there is no published literature in this area. Our findings can be compared to the usability of mobile apps using the SUS questionnaire, as numerous studies have been published in this field [18, 30, 34]. The mean usability score in our study was comparable to the mean usability scores found in two local studies, which were a mobile app for colorectal community education (72.9 [SD ± 11.5]) [31] and *Gigiku Sihat*, a mobile app for diet and dental hygiene (77.0 [SD ± 14.18]) [32]. The mean usability score in this study was also comparable to that in the study by Lim et al., measuring the usability of a self-management smartphone diary tool in patients with high cardiovascular risks (69.8 [SD ± 12.9]) [35].

The majority of participants agreed that they liked the physical appearance of the booklet, that all seven sections were simple to comprehend and that the majority of the doctors (88.5%) provided instructions on how to use the booklet. Nevertheless, only 75.7% used the booklet at home. Future studies should explore the barriers to using the booklet at home, as nearly a quarter of the patients in this study did not use the booklet to self-manage their conditions at home as intended.

Five factors were found to be significantly associated with the good usability of the EMPOWER-SUSTAIN Global Cardiovascular Risks Self-Management Booklet^©^ among individuals with MetS in our study, namely, those with high education levels (secondary and tertiary education), those who used the booklet daily or weekly at home, and those who had social support to use the booklet. Patients with higher education levels, secondary education (AOR 2.46, 95% CI 1.04, 5.83] and tertiary education (AOR 2.49, 95% CI 1.04, 5.96), had significantly higher odds of having good usability of the booklet compared to those with low education. This finding is consistent with several other studies [36, 37] showing that the mean usability scores of eHealth apps were significantly higher among patients with a higher level of education. These results may be attributable to the fact that those with a higher education level have more knowledge and greater access to the necessary information, and they are able to understand, memorize, and apply the information better than those with a lower level of education [38, 39].

Patients who used the booklet at home weekly (AOR 2.94, 95% CI 1.63, 5.33) or daily (AOR 2.73, 95% CI 1.09, 6.85) had significantly higher odds of having good usability of the booklet compared with those who did not use it at home. This finding is consistent with a usability study of a mobile app, DIABETEYAR, that assists diabetes patients in managing their self-care activities, in which the mean usability score was positively associated with increased weekly usage [36]. Therefore, healthcare professionals, especially primary care physicians, should encourage their patients to utilize the self-management booklet at home at least weekly to improve its usability.

Patients who received social support from their family members and friends had higher odds (AOR 1.64, 95% CI 1.02, 2.64) of having good usability of the booklet than those who did not receive social support. A review by Reiners et al. regarding factors influencing the use of eHealth in people with chronic diseases showed that including family members plays a crucial role in implementing and utilising eHealth [37]. Multiple-person households have been shown to positively influence the use of eHealth, where elderly individuals who struggle with technology are frequently assisted by family members [37]. Therefore, primary care physicians should promote participation and support from patients’ family members and friends to maximize the usability of this self-management booklet. Such activities will encourage patients to share their knowledge and skills in self-management of their condition with those around them.

### Strengths and limitations

A major strength of this study is that it addresses the gap in the literature where research on the usability of a self-management booklet in primary care is nonexistent. Despite the rapid growth in digital health, paper-based self-management booklets remain relevant as they are more accessible to a broader range of patients, including those who might have difficulties handling digital technologies or have limited internet access [18]. Another strength of the study is that the data set is of high quality, as there were no missing values. Nonetheless, this study has several limitations. Firstly, this study may be susceptible to reporting bias, particularly obsequiousness bias. The participants may answer the questionnaire in a manner they believe would please their doctors, as this booklet serves as an interaction tool between them and their doctors to monitor the progress of their disease. Secondly, this study was conducted at a university primary care clinic where most participants were Malays. The Chinese and Indian ethnic groups were underrepresented in this study. Therefore, the findings may not be generalizable to other primary care clinics in Malaysia with a multiracial population. Thirdly, several variables included in the MLogR have an extreme proportion (> 80% in one group), such as ethnicity and education levels, and the confidence intervals are relatively wide. This may have an effect on the final model, and therefore the findings should be cautiously interpreted given this limitation. Finally, this study did not explore other potential factors or barriers that may affect the usability of a self-management booklet, such as health literacy and self-efficacy. Therefore, the result of the MLogR is limited to the variables included in this study and must be interpreted within the context of this study.

### Implications for future research and clinical practice

The usability of the EMPOWER-SUSTAIN Global Cardiovascular Risks Self-Management Booklet^©^ among patients with MetS in our primary care clinic is established in this study, supporting its widespread utilization as an empowerment tool to aid productive interaction between doctors, nurses and patients with MetS. This joint health decision-making process will empower patients to improve their health outcomes, which will eventually prevent CVD complications such as heart attack and stroke. This study also established that five factors were significantly associated with the usability of the booklet. Primary care physicians should focus on those with lower education, as they may not understand the content of the booklet, as only those with higher education levels were found to have better usability of the booklet. Primary care physicians should also encourage their patients to utilize the self-management booklet at home at least weekly to improve its usability. Encouraging participation and support from patients’ family members and friends when using the booklet at home is also vital to maximizing its usability. Future research should also be conducted that includes other factors or barriers that may influence the usability of a self-management booklet, such as health literacy and self-efficacy. Future studies should also involve patients from various ethnic groups from other primary care clinics in Malaysia.

This booklet has recently been converted into a mobile app, and its development, design, utility and usability testing has been published elsewhere [40]. Further research will be conducted to evaluate this mobile app's effectiveness in changing lifestyle (diet and exercise) and improving clinical outcomes [15].

## Conclusions

In conclusion, this study found that most patients with MetS had good usability of the self-management booklet. High education levels (secondary and tertiary education), using the booklet daily or weekly at home, and having social support to use the booklet were identified as significant factors of good usability. Despite its limitations, this study is the only one that has determined the usability of a self-management booklet and its associated factors among patients with MetS in the Malaysian primary care setting. The findings of this study support the widespread use of this booklet as an empowerment tool to aid productive interaction between primary care providers and patients with MetS. This patient-centered decision-making process will empower patients to improve their health outcomes, eventually preventing CVD complications. Focus should be given to those with lower education to improve the usability of the booklet in this group. Further research is needed to evaluate important factors influencing the usability of a self-management tool, such as health literacy and self-efficacy.

### Supplementary Information

**Additional file 1.** The EMPOWER-SUSTAIN Usability Questionnaire (E-SUQ*©*) Malay Version.

## Data Availability

Data are kept at the Department of Primary Care Medicine, Universiti Teknologi MARA, in Selangor, Malaysia. Data will be shared upon request and subject to data protection regulations.

## Abbreviations

AOR: Adjusted odd ratio
BMI: Body mass index
BP: Blood pressure
CCM: Chronic Care Model
CI: Confidence interval
CVD: Cardiovascular diseases
DM: Diabetes mellitus
E-SUQ: EMPOWER—SUSTAIN Usability Questionnaire
FPG: Fasting plasma glucose
FSL: Fasting serum lipid
HDL-c: High-density lipoprotein cholesterol
HTN: Hypertension
LDL-c: Low-density lipoprotein cholesterol
MetS: Metabolic syndrome
MLogR: Multiple logistic regressions
MYR: Malaysian Ringgit
OR: Odd ratio
SD: Standard deviation
SLogR: Simple logistic regression
SUS: System Usability Scale
TC: Total cholesterol
TG: Triglycerides
WC: Waist circumference
