# Supplementary material for: Factors associated with usability of the EMPOWER-SUSTAIN Global Cardiovascular Risks Self-Management Booklet© among individuals with metabolic syndrome in primary care: a cross-sectional study
Source: BMC Prim Care. 2024 Feb 3;25:51. doi: 10.1186/s12875-024-02281-z (PMC10837927; doi:10.1186/s12875-024-02281-z)
Supplement: Supplementary file 1 — Additional file 1. The EMPOWER-SUSTAIN Usability Questionnaire (E-SUQ©) Malay Version. [file 12875_2024_2281_MOESM1_ESM.pdf]

# The EMPOWER-SUSTAIN Usability Questionnaire (E-SUQ©)– Malay Version

**Bagi soalan di bawah, sila tandakan jawapan yang paling sesuai berdasarkan skala seperti berikut.**

| | 1 | 2 | 3 | 4 | 5 | |
|---|---|---|---|---|---|---|
| Sangat tidak bersetuju | ← | | | | → | Sangat bersetuju |

| | | 1 | 2 | 3 | 4 | 5 |
|---|---|---|---|---|---|---|
| 1. | Saya rasa saya akan menggunakan buku pengawasan kendiri ini dengan kerap. | | | | | |
| 2. | Saya rasa buku pengawasan kendiri ini adalah buku yang rumit. | | | | | |
| 3. | Saya rasa buku pengawasan kendiri ini mudah untuk digunakan. | | | | | |
| 4. | Saya memerlukan bantuan daripada orang lain untuk menggunakan buku pengawasan kendiri ini. | | | | | |
| 5. | Saya mendapati isi kandungan buku pengawasan kendiri ini→ berhubungkait antara satu sama lain (Contoh: Faktor risiko kardiovaskular → Keputusan ujian darah → Pengurusan berat badan → Tabiat merokok → Kawalan tekanan darah/gula Senarai ubatan) | | | | | |
| 6. | Saya mendapati kandungan dalam buku pengawasan kendiri ini tidak konsisten antara satu sama lain. | | | | | |
| 7. | Saya berpendapat bahawa cara penggunaan buku pengawasan kendiri ini mudah dipelajari. | | | | | |
| 8. | Saya mendapati buku pengawasan kendiri ini leceh untuk digunakan. | | | | | |
| 9. | Saya berasa yakin menggunakan buku pengawasan kendiri ini. | | | | | |

| | | | | | | |
|---|---|---|---|---|---|---|
| 10. | Saya perlu belajar banyak perkara sebelum boleh menggunakan buku pengawasan kendiri ini. | | | | | |

Permission to use the E-SUQ© was obtained from the questionnaire developer.
